# Influence of the Composition on the Compressive Behaviour of a Semi-Metallic Brake-Pad Material

**DOI:** 10.3390/ma15227911

**Published:** 2022-11-09

**Authors:** Itziar Serrano-Munoz, Vincent Magnier, Florent Brunel, Philippe Dufrenoy

**Affiliations:** Université de Lille 1, CNRS, Centrale Lille, UMR 9013-LaMcube-Laboratoire de Mécanique, Multiphysique, Multiéchelle, F-59000 Lille, France

**Keywords:** semi-metallic brake-pad material, powder metallurgy, components influence, graphite induced hysteresis, digital image correlation (DIC), compression testing

## Abstract

The contact interface between the rotation and static part of a friction brake is central to the optimal functioning of the brake system due to the occurrence of heat dissipation, mechanical interaction and thermal exchanges. Generally, braking performances are evaluated by the energetic efficiency and wear rates of the contact surface. However, the compressive behaviour of the contact materials has also a significant contribution to the overall performances. In this work, the meso- and microscopic compressive behaviour of a sintered semi-metallic brake-pad material is investigated mainly via compression testing coupled with Digital Image Correlation (DIC) technique, as well as optical and scanning electron microscopy (SEM) analysis. The composition of a reference material (RM) is simplified to a selection of nine components, as opposed to up to thirty components typically used in commercial brake-pad materials. The retained components are considered as the most crucial for safe-operating performances. At the studied stress levels, the RM material is flexible (E = 5330 MPa), deformable (E${}_{zz-plastic}$ = −0.21%), and exhibits hysteresis loops. Subsequently, the contribution to the mechanical response of each individual component is investigated by producing the so-called dissociated materials, where the number of components is, at a time, further reduced. It is observed that the macroscopic behaviour is mainly controlled by the content (i.e., size distribution, shape and nature) of graphite particles, and that the hysteresis is only related to one of the two types of graphite used (G2 particles). Moreover, RM containing 13 wt% of G2 particles embedded in a relatively soft matrix (10.86 GPa) is able to increase the hysteresis (by 35%) when compared to the dissociated material containing 20 wt% of G2 particles which is embedded in a stiffer matrix (E = 106 GPa).

## 1. Introduction

Friction brake-pads are critical functional parts of the transportation industry that must assure a safe brake action under very wide operational conditions [1,2,3,4]. As such, brake-pads must comply with as many requirements as: adequate friction coefficient, good heat conductivity and low fade, durability, resistance to corrosion, stable friction, ease to maintenance, low noise (i.e., reduction of squeal phenomena) and wear, light weight and cost-effective manufacturing. Moreover, complex multiscale and mechano-chemical interactions (which might occur simultaneously) are involved during the braking process, where completely new chemical compounds are formed at the friction interface.

To be able to comply with all the above requirements, friction materials are design as heterogeneous composites that can contain up to thirty components. These components can be categorized into four main classes of: (i) abrasives (e.g., SiO${}_{2}$, ZrSiO${}_{4}$), to increase friction and control the build-up of friction films; (ii) friction modifiers to lubricate or help control interfacial films by reacting with oxygen (e.g., graphite particles, Cu or metal oxides); (iii) fillers and reinforcements to increase compactness (e.g., fibres, barytes); and (iv) binder materials to form the friction material matrix (e.g., phenolic resin, or metallic sintered powders). Hence, a stringent control of the formulation is imperative, as the slightest variation in components parameters (i.e., size, shape and composition) can lead to considerable changes in brake performances.

The advent of modern fast-moving vehicles has highlighted the necessity for new approaches enabling the further and faster development (while maintaining the highest safety standards) of new formulations to address the new braking-performance requirements (i.e., higher braking pressures applied to assemblies, and higher energy dissipation performances) and changing environmental legislations. For instance, the composition of friction materials has been subjected in recent years [5,6,7] to considerable debate because of the toxic impact of copper on the environment (e.g., killing invertebrates and algae, and harming fish), as well as the copper contribution to cause adverse health effects in humans (e.g., causing myocardial infraction and lung diseases [8]). In fact, due to the adoption of new regulations focusing on environmental protection [9,10], the automotive industry faces a <0.5 weight percent (wt%) reduction in copper content by 2025.

The replacement of copper is not a straightforward process. It is generally accepted that the presence of copper is crucial for the development of friction layers and optimal heat flow. Its removal leads to increase wear rates and higher friction coefficients, which subsequently facilitates the production and emission of airborne particulate matter [11]. Furthermore, it has been observed that Fe-based (i.e., iron has the highest weight percentage (wt%) in the formulation) semi-metallic brake-pad materials exhibit lower friction performances than those with Cu-based metallic matrix [12]. The replacement of copper requires a thorough reconsideration of brake formulations to maintain suitable friction levels and durability (i.e., wear rates).

Despite the fact that friction materials must be continuously improved and evolved, the evaluation of process-microstructure-properties relationships remains a purely empirical process based on time-consuming and expensive bench tests. There is a lack of knowledge in the open literature regarding the way components influence brake response and the way the components influence each other. To be able to improve cost-effectiveness of the development and evaluation processes of brake-pads, realistic numerical tools are needed. These tools can provide a predictive understanding of the change in performances caused by modifications in the composition.

For the numerical simulations to be realistic, the friction material behaviour must be first understood in conditions as similar as possible to the in-service ones. This characterisation is a major challenge because it requires knowledge of the mechanical (dumping effects and surfaces contact), thermal (temperature built-up and dissipation), tribological (friction layer development), and dynamic (squeal generation) performances, as well as the elucidation of how these performances evolve during different duty cycles. Moreover, the friction and durability parameters are highly dependent on the development of an optimal contact surface between the rotating disk and the brake-pad. In turn, the development of an optimal surface contact requires the use of materials with reduced stiffness to avoid localisation of the wear, promoting a *global* wear. Nevertheless, the compressive behaviour is rarely investigated in comparison to wear performances (see references [13,14], as one of the few examples of investigation of the compressive behaviour of brake pads).

The present study intends to rationalize the role of components in the local deformation mechanisms under compressive loading for the case of a semi-metallic brake-pad material in its as-fabricated condition. The endgame is to obtain realistic behavioural laws of the as-fabricated state to be deployed at the start of numerical simulations of the braking process. In order to *simplify* the characterization task, the formulation of the studied material was reduced to nine components (those considered the most influential to brake performances). In addition, to be able to fully understand the role played by each one of the components, a dissociation strategy was carried out. This strategy consists in producing four types of dissociated materials, which contain some of the nine original components. Light optical microscopy (OM) and Scanning Electron Microscopy (SEM) is used for microstructural characterization at the meso and microscale. A 2D-Digital Image Correlation (DIC, only one camera recording) technique is used during the compression testing to measure the displacement fields of the materials at different loading stages. The most relevant results are presented and the discussion is focused on the understanding of how the difference in formulation (in terms of differences in components content) affects the local deformation behaviour.

## 2. Materials and Methods

### 2.1. Materials

Five different types of materials were investigated in this work. Their formulation and manufacturing processes are described below and a summary of the compositions (in weight percentage, wt%) is given in Table 1.

#### 2.1.1. Reference Material

The studied reference material (RM) is a semi-metallic brake-pad material provided by FLERTEX Transport company (Gennevilliers, France), which is principally used in the railway industry. Its composition was reduced to only those nine components that are considered essential to brake performances. These components are classified into (i) metallic powders (iron and copper) and fillers (ii) ceramic particles (SiC and ZrSiO${}_{4}$), and (iii) two types of graphite particles (labelled as G1 and G2). The weight percentages (wt%) of the nine components are given in Table 1. In this table, the fillers column accounts for three different components. The chemistry of the fillers is proprietary to FLERTEX and cannot be disclosed. The RM is considered as a Fe-based material. The size distributions are given in Table 2.

The fabrication process consisted in: (i) powder preparation and mixing of raw materials, (ii) compression of powders (up to 1000 tons, along the Z axis, which is also the in-service working direction) in a shaped mould to produce green compacts, and (iii) sintering in a controlled atmosphere furnace during 8 h at ∼1100 °C. The material was manufactured to their final shape. Hereafter, this is named the as-fabricated condition.

Special attention is given to the graphites particles in this work. This is because a literature review indicates that they can be considered as the main responsible for the hysteresis phenomenon [15]. G1 (Graphite “4020”, Mersen group, Gennevilliers, France) is a secondary synthetic graphite, and it can be argued that this component is not a pure graphite. G2 is a primary synthetic graphite (has higher purity and crystallinity, TIMREX${}^{®}$ KS 300-1250) and was provided by Imerys Graphite & Carbon Company (Paris, France). These two particles yield similar grey levels when examined via microscopy and cannot be unambiguously identified. Thus, microscopical classification was based on the size and shape: G1 particles are platelet-like, whereas G2 particles possess an increased sphericity and size, which allowed to observe some of the G2 particles with the naked eye.

#### 2.1.2. Dissociated Materials

**Figure 1:**
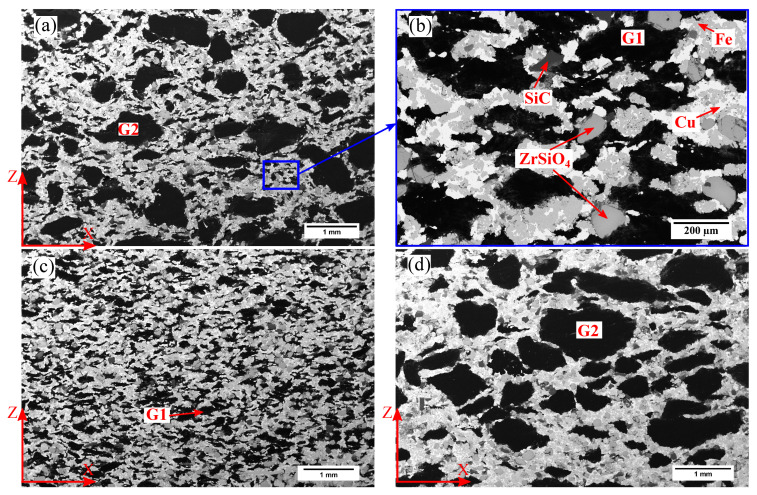
SEM Backscattered images of the (**a**) microstructure of the RM material at the macroscale. (**b**) microstructure of RM material at the mesoscale, (**c**) microstructure of MMCSG1 material at the macroscale, and (**d**) microstructure of MMCSG2 material at the macroscale.

The formulations of the dissociated materials were chosen so only one component is added at a time, from the basic metal matrix, all the way up to the RM. The manufacturing process is kept the same to that introduced above. The particle size distributions were also kept the same and only the weight percentages are modified. The Metallic-Matrix (MM) material, containing pure Fe and Cu powders and fillers (see Figure 1b), as well as the Metallic-Matrix-Ceramics (MMCs) material, containing pure Fe and Cu powders, fillers, SiC and ZrSiO${}_{4}$, were produced by respectively adapting the wt% values so the original wt% ratio (e.g., 1Fe:0.75Cu:0.28Fillers for the case of MM) was maintained (see Table 1).

The formulation of the Metallic-Matrix-Ceramics-G1 (MMCsG1) material is that of RM material except for the fact that it only contains one type of graphite (G1 particles) at 20 wt% in order to maintain the same ratio of graphite within the metallic matrix (i.e., adding the 13% of G2 to the original 7% G1 contained in RM). Likewise, the Metallic-Matrix-Ceramics-G2 (MMCsG2) material only contains G2 particles at 20 wt%.

### 2.2. Methods

#### 2.2.1. Optical Microscope and SEM Observations

Regardless the material (i.e., RM or dissociated materials), the same metallographic specimen preparation procedure was used. It consisted of rough and fine grinding from P500 down to P4000 grit papers (with intermediate P1200 and P2500 papers), where the use of water was avoided. This was followed by polishing on cloth using a diamond particle suspension of 3 μm. Final polishing was performed using MastMet-2 non-crystallizing colloidal silica suspension of 0.02 μm.

Samples of RM, MM and MMCs materials were prepared for optical microscopy and sets of five images per material (ten for RM material, as graphite presence reduces Cu statistics per image) were taken at the microscale (×20). These sets of images were analysed using the plugin “Analyze Particles” of Fiji${}^{®}$ 1.53t free software [16]. Prior to SEM (Hitachi-3600N, Hitachi High-Tech, Tokyo, Japan) observations, polished samples of RM, MMCsG1 and MMCsG2 materials were etched with Nital solution for no longer than 5 s.

#### 2.2.2. Compression Testing

All five materials were tested in their as-fabricated condition using the testing standard DIN 50106 [17]. Compressive tests were performed using an INSTRON-5500 machine (Instron Group®, Norwood, MA, USA) equipped with a load cell of 50 kN. The loading rate was 0.01 mm/s. The loading direction corresponds to the Z axis (i.e., in-service working direction). The tested samples are cubes of 20 × 20 × 20 mm${}^{3}$; this volume was considered large enough to be representative of the macroscopic behaviour of RM, MMCsG1, and MMCsG2 materials. In the case of MM and MMCs materials, the sample size was reduced to 5 × 5 × 10 mm${}^{3}$. The top and bottom surfaces were rectified to ensure a good parallelism, and one of the lateral surfaces was ground down to P1200 grit paper. This surface was completely sprayed in black to create the background before applying an airbrushed white speckle pattern prior to digital image correlation (DIC).

Samples were typically preloaded at $\sigma_{min}$ = 1 MPa and tests start with $\sigma_{max}$ = 3 MPa, performing five to ten loops (Δσ = 2 MPa). Subsequently, the maximum load was increased to $\sigma_{max}$ = 5 MPa, $\sigma_{max}$ = 10 MPa, $\sigma_{max}$ = 15 MPa, and finally $\sigma_{max}$ = 20 MPa, where the looping process at every load increase is replicated. For the sake of clarity, only the results at $\sigma_{max}$ = 20 MPa are used in what follows to compare the different compressive behaviour of RM, MMCSG1, and MMCSG2 materials. In addition, the RM was tested directly between 1 and 20 MPa.

During the compression testing, two optics fibres illuminated the sprayed surface, while a XIMEA camera with 4.0 Mega pixels (2048 × 2048 pixels) recorded images of the whole sample surface (∼20 × 20 mm${}^{2}$) at every 500 ms. The image resolution was about 300 μm. Therefore, only the influence of G2 particles could be studied with this method. DIC was performed between the preload image and the deformed images corresponding to each loading step. YaDICs free software [18] was used to resolve the displacement and strain fields. This software used the entire image as a sampling grid (i.e., total sampling) with bicubic interpolation being used for the grey level evaluation at non-integer coordinates. The Optical Flow elastic transform based on Finite Element Model kinematics (OF-FEM) method was used with discretized pixel subsets of 14 × 14 pixels. The OF-FEM approach reduces measurement uncertainties because they require continuity of the found displacement field throughout the region of interest [19]. The accuracy of this method was already verified by comparing DIC and strain gauge results obtained simultaneously out of the same sample [20].

## 3. Results and Discussion

### 3.1. Microstructure

**Figure 2:**
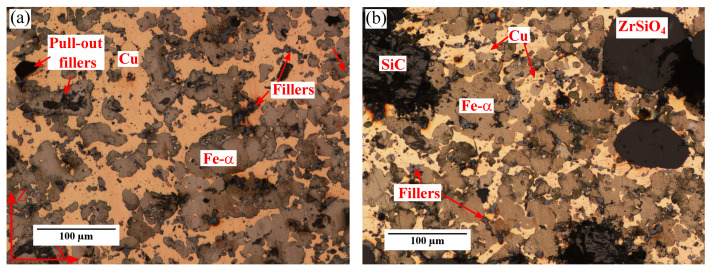
(**a**) Optical microscopy micrograph at ×20 of the MM material after Nital etching. (**b**) Optical microscopy micrograph at ×20 of the MMCs material after Nital etching.

G2 particles are the largest and some of them can be observed with the naked eye. Therefore, in this study, they are considered as being part of the macroscale length (see Figure 1a,d). Second in size are G1 and ceramics particles (see Figure 1b,c). They have intermediate sizes and are considered as part of the mesoscale length of the material. Finally, the metallic powders and some fillers are the smallest and belong to the microscale length (Figure 2).

### 3.2. Equivalence of the Studied Materials

Even though the fabrication process is kept the same for all materials, their equivalence needs to be investigated. This is because the mixing of raw materials can be affected depending on the number of components that are being blended. The size (using Feret’s diameter; i.e., the longest distance between any two points along the selection boundary, also known as maximum caliper.) and shape (using Circularity = 4π×[Area]/[Perimeter]${}^{2}$, which ranges from 0 (infinitely elongated polygon) to 1 (perfect circle)) of Cu phase are measured in MM, MMCs and RM materials using optical micrographs at ×20 magnification. The results (average values, see Table 3) indicate that the addition of ceramic particles (MMCs), and ceramic and graphite particles (RM) during the mixing of raw materials does not notably alter the morphology of the Fe-based metallic matrix.

Another key issue is the modification of the metallic matrix during the sintering. It has been reported that C originating from SiC particles can be dissolved in the Fe-based matrix. This typically leads to the formation of pearlite phase. For instance, for a Fe-SiC (5 wt%) metal matrix composite sintered during 2 h, some reaction between the SiC particles and Fe was reported to occur at 1000 °C [21]. However, this is not the case for the MMCs material. Neither pearlite formation, nor a decrease in size of SiC (or ZrSiO${}_{4}$) particles is observed. Moreover, when compared, the etched microstructures of MM and MMCs materials looks similar (see Figure 2). Hence, it is considered that the presence of ceramics does not induce the formation of new phases in the Fe-α matrix.

**Figure 3:**
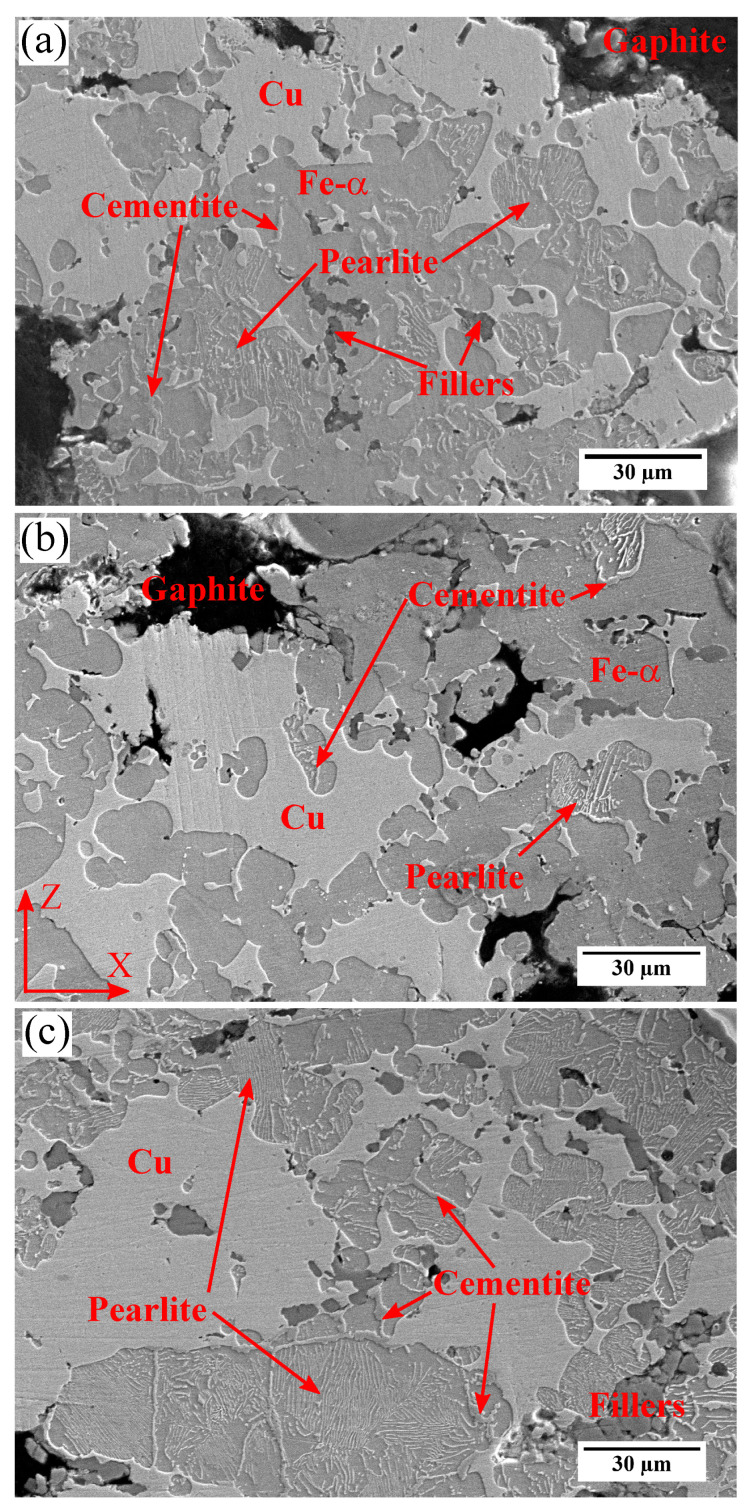
Secondary Electrons SE-SEM images of the (**a**) microstructure of RM material at the microscale length, (**b**) microstructure of MMCsG1 material at the microscale length, (**c**) microstructure of MMCsG2 material at the microscale length.

On the contrary, changing the mass percentages of graphite particles does affect the Fe-α matrix. The microstructure of RM material (Figure 3a) exhibits some unevenly distributed clusters of thin pearlite with occasional small platelets of cementite. Note that in this study the distinction between cementite and pearlite is based on the size: the clusters where the cementite platelets are very thin and barely distinguishable are labelled as pearlite, whereas the cementite label is given to those platelets that are *thick* enough to be clearly distinguishable from the ferrite matrix. For MMCsG1 material, the presence of pearlite/cementite is rarely observed (Figure 3b). Finally, Figure 3c shows that fine pearlite phase is dominant in MMCsG2 material with occasional islands of Fe-α phase also being observed. The addition of graphite is a standard practice in powder metallurgy to induce strengthening and hardening [22]. The nature (fine or coarse), spacing and amount of pearlite depends on the processing conditions. For the studied materials, it can be considered that G2 particles are the main responsible for carbon diffusion in Fe-α and that increasing their content (from 13% to 20 wt%) increases the amount of pearlite/cementite. However, the determination of the amount of carbon diffusing in iron during the sintering is out of the scope of this study. Overall, the metallic matrix of the three materials can be considered equivalent, whereas the metallic matrix of MMCsG2 is expected to yield relatively higher stiffness. Note that the exact amount of increase provided by the presence of perlite/cementite is undetermined. Furthermore, it has been reported that the temperatures reached during braking operations can cause further dissolution of carbon into the metallic matrix [14].

### 3.3. Compressive Behaviour

#### 3.3.1. RM Material

**Figure 4:**
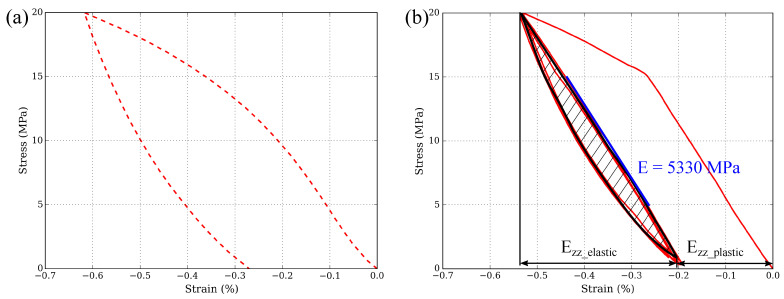
(**a**) Compressive stress-strain (E${}_{zz}$) curve of a RM specimen directly tested between 0 and 20 MPa. (**b**) Compressive stress-strain (E${}_{zz}$) curve of a RM specimen after prior testing between 0 and 15 MPa. The area of the hysteresis loop is indicated in black.

Figure 4a shows the stress-strain curve of RM material when tested directly between 0 and 20 MPa. During the loading, the stress-strain curve tends to decrease continually and, as a result, this material does not exhibit a true elastic behaviour. The unloading is also non-linear.

The stress-strain curve changes when the material is compressed at 20 MPa after a previous test at 15 MPa (see Figure 4b). Here, the loading is mainly linear until reaching 15 MPa where the plastic deformation starts. Because of the non-linear unloading, it was decided to measure the modulus of elasticity at the second loading, between 5 and 15 MPa (E = 5330 MPa). For comparison purposes, the E${}_{zzelastic}$ and E${}_{zzplastic}$ values are taken at the preload (σ = 1 MPa). The induced plastic strain is E${}_{zzplastic}$ = −0.21% and the elastic part renders E${}_{zzelastic}$ = −0.34%. Note that only E${}_{zz}$ values are shown instead of E${}_{von-Mises}$ results because E${}_{zz}$ values are considerably higher than E${}_{xx}$ and E${}_{xz}$ ones. Hence, it is considered that the Z-axis dominates the onset of the most relevant mechanisms. The hysteresis generated within one loop is measured using the hatched surface shown in Figure 4b, which gives an area value of 0.77 MPa×%.

**Figure 5:**
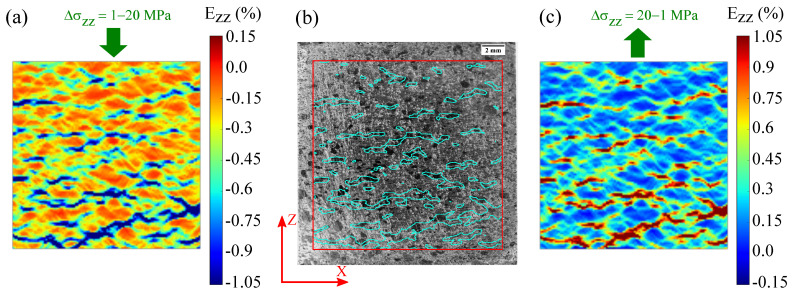
(**a**) E${}_{zz}$ strain field of RM, obtained using DIC, of the elastic part of the loading between 1 and 20 MPa. (**b**) Image recorded with a XIMEA camera, prior to the deposition of the speckle pattern (20 × 20 × 20 mm${}^{3}$). Only the G2 particles (in darker grey values) can be distinguished in this image. The square in red indicates the region of interest (ROI) where DIC is performed. The cyan outlines, which are obtained from Figure 5a, show the outline of the regions where strain values correspond to −0.6% (**c**) E${}_{zz}$ strain field of the elastic part of the unloading between 1 and 20 MPa.

Figure 5a shows that the strain distribution throughout the specimen surface during the elastic loading is heterogeneous. There are some regions showing high strain localisation, with values between −0.6 and −1.05% (shown in blue). When compared with the microstructure, it is observed that these blue regions mostly correlate with the presence of G2 particles (see Figure 5b). The strain distribution during the unloading is also heterogeneous (Figure 5c). Moreover, the strain field during the unloading is the opposite to that generated during the loading; the regions with high strain localisation during the loading are the most unstrained during the unloading (i.e., region in red in Figure 5c).

#### 3.3.2. Dissociated Materials

**Figure 6:**
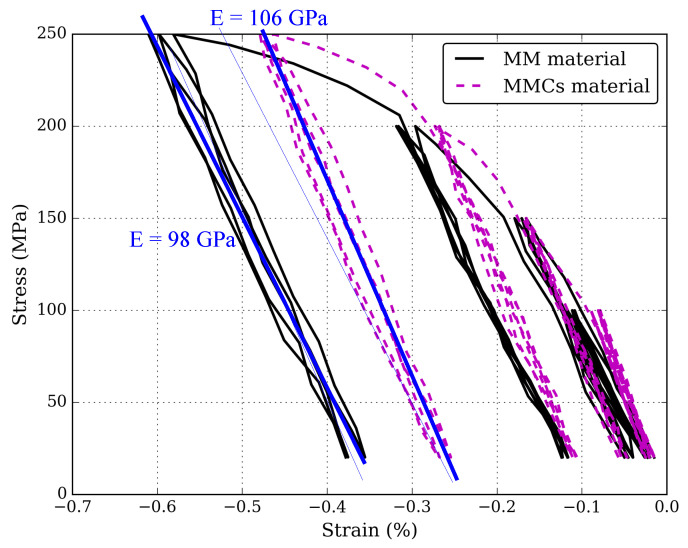
Plots describing the compressive stress-strain (E${}_{zz}$) curve of MM (in black) and MMCs (in dashed magenta) materials.

Figure 6 shows the stress-strain curve of the MM material (in black). Here, the material is compressed between 0 and 250 MPa with intermediate looping at 100, 150 and 200 MPa. The elastic modulus is E = 98 GPa and the yield limit is $\sigma_{YS}\approx$ 120 MPa. The elastic modulus increases (by ≈ 10 GPa to E = 106 GPa) when ceramics are added, although, due to local stress concentration induced by the ceramics, the yield limit decreases ($\sigma_{YS}$ ≈ 100 MPa). Furthermore, the MMCs material requires higher stress values to produce similar plastic deformation, and no significant hysteresis is observed in both MM and MMCs materials.

**Figure 7:**
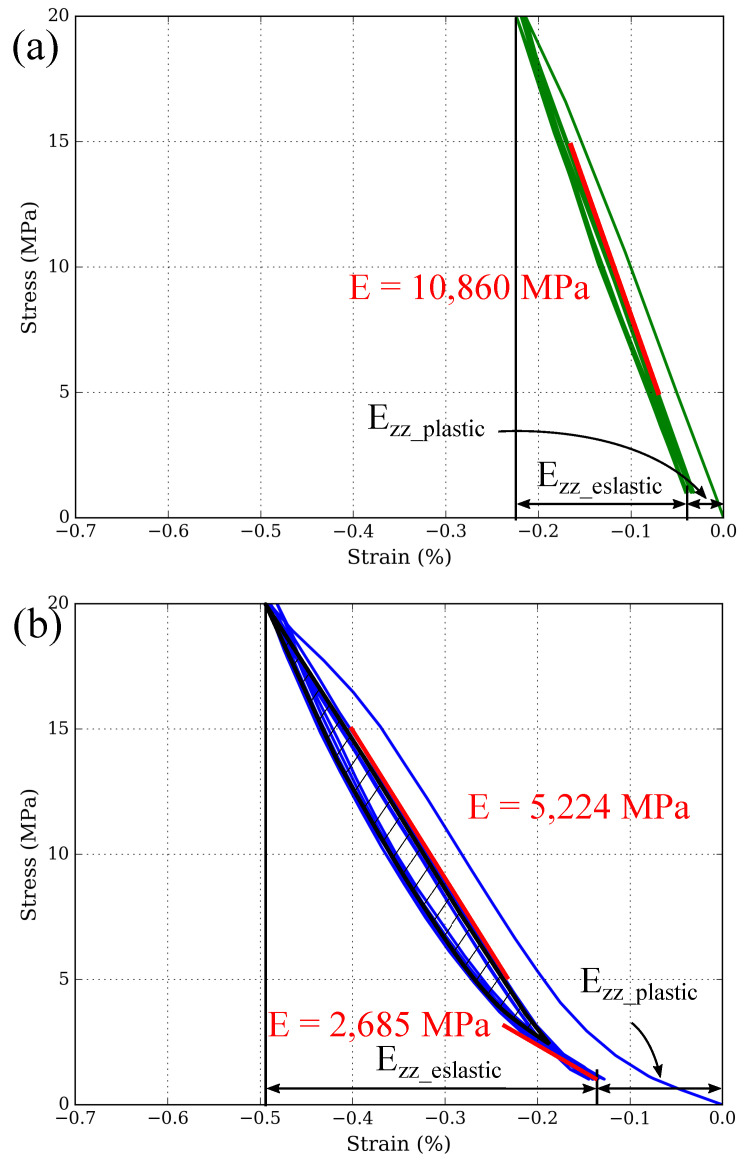
(**a**) Compressive stress-strain (E${}_{zz}$) curve of MMCsG1 material after prior testing between 0 and 15 MPa. (**b**) Compressive stress-strain (E${}_{zz}$) curve of MMCsG2 material after prior testing between 0 and 15 MPa. The area of the hysteresis loop is indicated in black.

Figure 7 shows that the compressive behaviour significantly varies depending on the graphite content. For MMCsG1 material (Figure 7a), the elastic modulus is E = 10,860 MPa, the plastic deformation E${}_{zzplastic}$ = −0.03%, the E${}_{zzelastic}$ = −0.2% and the hysteresis is so low that is considered negligible. The compressive behaviour of MMCsG2 material is more similar to that of RM: a two-fold stress-strain curve is developed, where the elastic modulus is E ≈ 2685 MPa at the end of the unloading (Δσ = 3–1 MPa) and E = 5224 MPa at the middle of the loading (Δσ = 5–15 MPa, Figure 7b). Also, the plastic strain is E${}_{zzplastic}$ = −0.14%, the E${}_{zzelastic}$ = −0.35%, and the hysteresis area is 0.5 MPa×%.

Thus, it can be concluded that G2 particles are the only component producing hysteresis. Interestingly, the RM is able to produce more hysteresis (0.77 against 0.5 MPa×%) even though the G2 content is lower in RM than in MMCsG2 (13 against 20 wt%). RM also produces more E${}_{zzplastic}$ while the E${}_{zzelastic}$ values are similar.

Regarding the strain localization, it is observed that the MMCsG1 material produces a homogeneous strain field both at the loading and unloading. Moreover, no differences are observed between the loading and the unloading. Nevertheless, it is suspected that some (non-resolved) strain localisation is produced by below resolution G1 particles. For the MMCsG2 material, the loading and unloading strain fields are similar to those shown in Figure 5 for RM.

### 3.4. Differences between the Investigated Materials

As mentioned in the Introduction, the principal reason to add graphite particles is because they provide lubrication during the braking process. Consequently, the size, shape and nature of graphite particles are chosen to comply with the lubrication requirements. Nevertheless, compressive testing results show that the presence of graphite particles controls the macroscopic compressive behaviour of RM, which, at this scale length, can be considered as a bi-component formed by G2 particles embedded in a metallic matrix. Hence, the content, size and shape of graphite particles can be tailored to fulfil both lubrication and mechanical response criteria; keeping in mind that good tribological performances are paramount for safety and must be always favoured. In fact, what is expected of the mechanical response of a brake-pad material is flexibility and low E values to favour a more homogeneous contact between the brake-pad and brake-disk surfaces, as well as some damping effects in order to reduce vibrations and noise.

**Figure 8:**
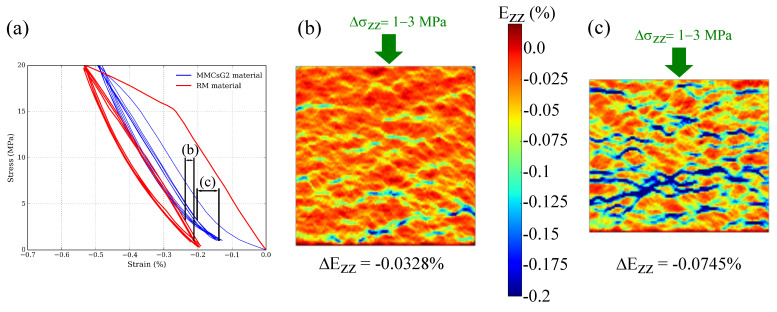
(**a**) Compressive stress-strain (E${}_{zz}$) curves of RM and MMCsG2 materials. (**b**) DIC E${}_{zz}$ image of the RM corresponding to the Δσ = 1–3 MPa loading within the loops. (**c**) DIC E${}_{zz}$ image of the MMCsG2 material corresponding to the Δσ = 1–3 MPa loading within the loops.

Given that only G2 particles are observed to produce hysteresis during the looping, the analysis on the differences in behaviour produced by the presence of graphite particles is focused on RM and MMCsG2 materials (Figure 8a). One of the main differences between these materials is the elastic behaviour at Δσ = 1–3 MPa of the loading part. At this early stress interval, the MMCsG2 material produces double the elastic modulus than that of RM. In fact, when the DIC results are compared (Figure 8b,c), it is observed that the strain localisation is more significant in the MMCsG2 material. Moreover, the MMCsG2 elastic modulus (E = 2685 MPa) at this stress interval is closer to that of synthetic graphite products (i.e., between E = 5000 and 10,000 MPa at room temperature [23]) than to the elastic modulus of the MMCs matrix (E = 106,000 MPa, Figure 6).

Assumably, the E${}_{zz}$ strain in the MMCsG2 material would be mainly accommodated by G2 particles during this first stage of compression. In other words, a higher content of G2 particles (from 13 to 20 wt%) increases the occurrence of particle clustering (i.e., the spacing between particles is expected to be reduced), which subsequently leads to the formation of regions where the volume fraction of graphite particles is considerably higher than that of the metallic matrix. Note that a detailed evaluation of the particle spacing is beyond the scope of this work and requires the use of 3D techniques such us microcomputed tomography.

**Figure 9:**
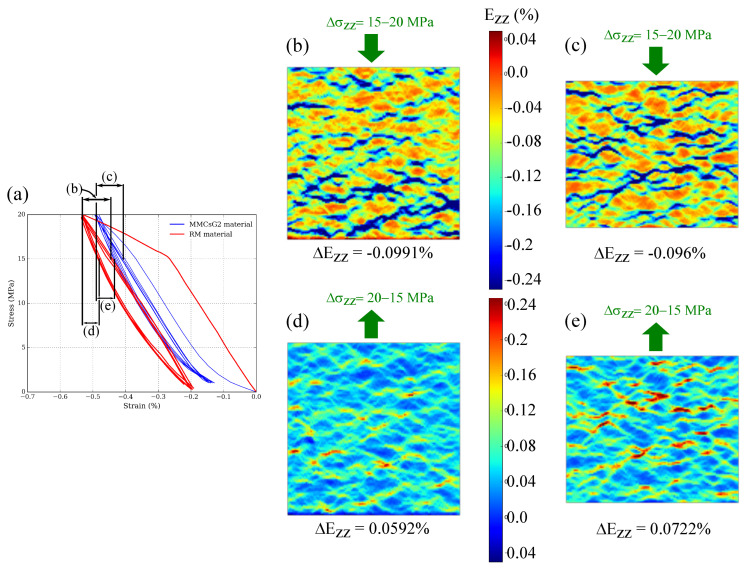
(**a**) Compressive stress-strain (E${}_{zz}$) curves of RM and MMCsG2 materials, where Δσ = 15–20 MPa and Δσ = 20–15 MPa elastic intervals are indicated with black arrows. (**b**) DIC E${}_{zz}$ image of the RM corresponding to the Δσ = 15–20 MPa loading within the loops. (**c**) DIC E${}_{zz}$ image of the MMCsG2 material corresponding to the Δσ = 15–20 MPa loading. (**d**) DIC E${}_{zz}$ image of the RM corresponding to the Δσ = 20–15 MPa unloading. (**e**) DIC E${}_{zz}$ image of the MMCsG2 material corresponding to the Δσ = 20–15 MPa unloading.

Another significant difference is the amount of hysteresis: 0.77 MPa×% for RM against 0.5 MPa×% for MMCsG2 material. During the loading, at the stress interval of Δσ = 15–20 MPa (see Figure 9a), the amount of E${}_{zz}$ strain produced by both materials is similar (ΔE${}_{zz}$≈−0.09%). The DIC strain fields of RM and MMCsG2 materials are also similar (see Figure 9b,c). However, both the E${}_{zz}$ strain and the DIC strain fields are different during the unloading at Δσ = 20–15 MPa interval. This is because the recovery is higher for MMCsG2 material: ΔE${}_{zz}$ ≈ 0.07% against ΔE${}_{zz}$ ≈ 0.06% for RM material. Furthermore, the DIC strain fields (Figure 9d,e) indicate that this difference is mainly due to the presence of highly strained regions (shown in blue during the loading, and turning to red during the unloading). As such, the strain recovery of MMCsG2 material in Δσ = 20–15 MPa interval should be higher than the recovery underwent by the RM.

The results introduced above are puzzling mainly because: (i) two different materials are producing almost identical results during the loading; nevertheless one would expect a higher amount of strain location in the MMCsG2 material induced by the higher content of G2; (ii) given that G2 particles are the main contributor to the development of hysteresis, one would expect that the amount of hysteresis produced by MMCsG2 material to be higher than that produced by RM.

**Figure 10:**
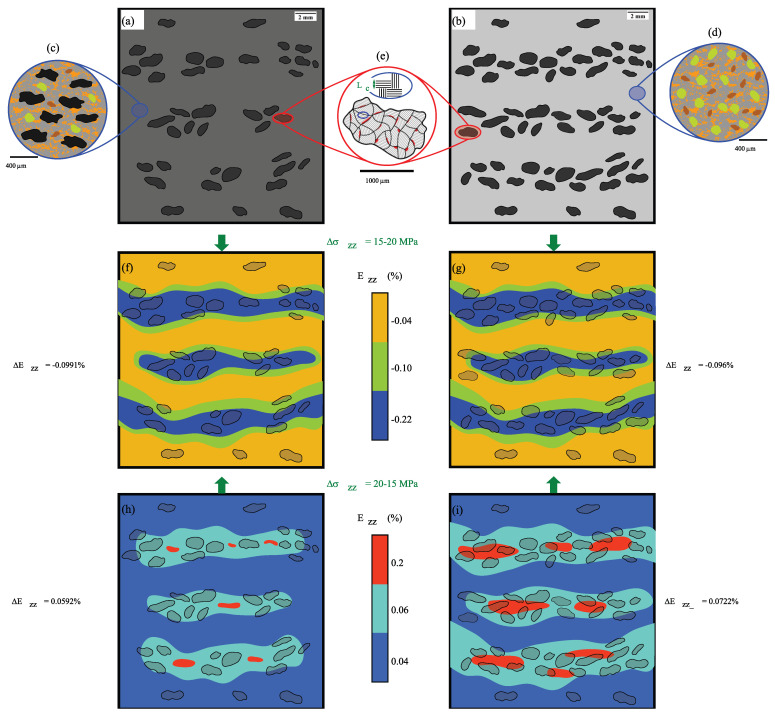
Schematic illustrations of the components at (**a**) the macroscale of RM, (**b**) the macroscale of MMCsG2 material, (**c**) the mesoscale of RM material, (**d**) the mesoscale of MMCsG2 material, and (**e**) the microscale of a G2 particle. Schematic illustrations of the compressive behaviour of (**f**) the RM during the loading interval of Δσ = 15–20 MPa, (**g**) the MMCsG2 material during the loading interval of Δσ = 15–20 MPa, (**h**) the RM during the unloading interval of Δσ = 20–15 MPa, and (**i**) the MMCsG2 material during the unloading interval of Δσ = 20–15 MPa.

As argued above, the RM contains 13 wt% of G2 particles (see Figure 10a), while the MMCsG2 material has a higher content (20 wt%, Figure 10b), which would induce an increased G2 particles interconnectivity. At the length scale at which the DIC measurements were performed, it can be assumed that the RM and MMCsG2 materials have a compressive response equivalent to that of a bi-component: i.e., G2 particles embedded in a matrix. In this context, the matrix of RM is expected to have a mechanical behaviour very similar to that of the MMCsG1 material (E = 10,860 MPa, Figure 10c), whereas the behaviour of MMCsG2 matrix would be considerably stiffer and similar to the mechanical behaviour exhibited by the MMCs material (E = 106 GPa, Figure 10d).

Graphite particles are a poly-crystalline materials made of several single crystals. The crystal structure consists of carbon atoms parallelly arranged in planar hexagonal networks [15,24]. This 2D-planar structure has strong covalent bonds between the atoms forming the layers (i.e., crystallites), although it forms weak van der Waals bonds between the layers of hexagons. The crystallite size is usually measured using the L${}_{C}$ parameter (see Figure 10e). Crystallite sizes larger than 100 nm are considered as macro-crystalline. The graphite particles used in brake-pad materials are chosen to be macro-crystalline in order to provide high thermal conductivity, lubricity and compressibility. These properties can be strongly influenced by the porosity typically contained within graphite particles, as shown in red in Figure 10e).

When G2 particles are compressed, the air and/or water contained within G2 intrinsic pores would be expelled most likely causing additional van der Waals bonds at the interfaces. Therefore, one explanation for the higher hysteresis observed in the RM could be that the higher flexibility of the matrix of this material leads to an increased bonding effect between the crystallites, implying that the G2 particles of RM accommodate higher strains due to higher localisation. Instead, the DIC results show that the strain localisation of both materials is almost identical (see Figure 10f,g). Thus, the bounding effect induced by compression is considered similar for both materials, which consequently means that the hysteresis should have to be higher for the MMCsG2 material.

Hence, it seems most likely for the compressive behaviour to be controlled by the matrix. The matrix of RM is more flexible and, when compressed at the same stress level, it accumulates double the plastic deformation when compared to the matrix of the MMCsG2 material (E${}_{zz-plastic}$ = −0.21% against E${}_{zz-plastic}$ = −0.14%). The similarity between the two materials loading response can be explained by the fact that the RM matrix is able to accommodate larger fractions of the highest strains (Figure 10f,g). Moreover, in comparison to the MMCsG2 material, the recovery produced by the matrix of RM in the early stages of unloading should be lower and more influenced by the bounding effect of G2 particles (Figure 10h,i). As such, the fact of possessing a more deformable matrix in the case of RM leads to the development of increased hysteresis even when using less amount of G2 particles.

Note that, apart from their tribological and mechanical role, graphite particles also work as an additive during the sintering inducing strengthening and hardening by the formation of pearlite/cementite (see Figure 3). It is uncertain if the strengthening of the metallic matrix (increased $\sigma_{YS}$ and ultimate tensile strength (UTS) can improve the braking performances. Higher hardness values most certainly have a deleterious effect by hampering the formation of friction layers and increasing wear rates of the braking system. Moreover, an increased C dissolution decreases the thermal conductivity of the Fe-matrix [25]. Thus, when tailoring the content of graphite particles in the formulation, attention must be also paid to the possible deleterious effects of increased C dissolution on the braking performances of Fe-based brake-pad materials.

## 4. Conclusions

The influence of the composition on the compressive behaviour of a simplified semi-metallic brake-pad material (RM) was studied and compared with four different dissociated materials (namely, MM, MMCs, MMCsG1, and MMCsG2). The following conclusions can be drawn:

1. Changing the content (i.e., size distribution, shape and nature) of graphite particles leads to considerable different mechanical responses.
2. The RM exhibits the highest plastic deformation at 20 MPa (E${}_{zz-plastic}$ = −0.21%). This is caused by the combined strain localization effect of ceramics, G1 and G2 particles.
3. The hysteresis observed in the RM can be related uniquely to the presence of G2 particles.
4. Higher levels of hysteresis can be produced with lower content of G2 particles if these particles are embedded is a more flexible matrix.
5. Changing the content and the type of graphite particles influences C dissolution in the metallic matrix. Higher content of G2 particles leads to higher C dissolution and therefore higher expected hardness and strength of the metallic matrix.

## Figures and Tables

**Table 1:** Summary of the formulation (in wt%) of all materials used in this work.

	Fe	Cu	Fillers	SiC	ZrSiO${}_{4}$	Graphite 1	Graphite 2
RM	34	26	10	2	8	7	13
MM	49	37	14	–	–	–	–
MMCs	43	32	12	3	10	–	–
MMCsG1	34	26	10	2	8	20	–
MMCsG2	34	26	10	2	8	–	20

**Table 2:** Size distribution, in μm, of six of the components used in the formulation of RM.

	Copper	Iron	SiC	ZrSiO${}_{4}$	G1	G2
Size (μm)	<100	<220	[20–260]	[80–320]	[50–500]	[300–1250]

**Table 3:** Size and shape measurements of Cu phase within the metallic matrix.

	MM	MMCs	RM
Cu Feret’s diameter (μm)	66.8 ± 3	51.1 ± 8	62.4 ± 5
Cu Circularity	0.56	0.53	0.55

## Data Availability

The datasets generated during and/or analysed during the current study are available from the corresponding author on reasonable request.

## Sample Availability

Authors agree to make materials supporting the results or analyses presented in their paper available upon reasonable request.

## Abbreviations

The following abbreviations are used in this manuscript:

RM	Reference material
MM	Metallic Matrix
MMCs	Metallic Matrix Ceramics
MMCsG1	Metallic Matrix Ceramics Graphite type 1
MMCsG2	Metallic Matrix Ceramics Graphite type 2
